# Predictors of COVID-19 actual vaccine uptake in Hong Kong: A longitudinal population-based survey

**DOI:** 10.1016/j.ssmph.2022.101130

**Published:** 2022-05-20

**Authors:** Elsie Yan, Daniel W.L. Lai, Haze K.L. Ng, Vincent W.P. Lee

**Affiliations:** a Department of Applied Social Sciences, The Hong Kong Polytechnic University, Hong Kong; b Faculty of Social Sciences, Hong Kong Baptist University, Hong Kong

**Keywords:** COVID-19, Pandemic, Vaccines, Vaccination, Health belief, Perception

## Abstract

**Purpose of the research:**

Identifying predictors of COVID-19 vaccine uptake decisions is central to the development of evidence-based strategies for promoting vaccination. This longitudinal study investigated the link between previous willingness to vaccinate and vaccine uptake decision, and examined potential predictors of vaccine uptake in Hong Kong.

**Methods:**

A longitudinal telephone survey study was conducted using a population-based sample of Chinese adult residents (≥18 years) in Hong Kong. Data were collected at two time points: T1 (December 2020–January 2021) and T2 (June–July 2021). Primary outcome was vaccine uptake status; whilst independent variables and covariates included socio-demographic factors, COVID-19 related experiences, health beliefs, and perception, as well as vaccine related perceptions.

**Results:**

Among the 1,003 participants, 23.7% had received a COVID-19 vaccine. Previous willingness to vaccinate did not predict vaccine uptake at later stage. Vaccine uptake by known others (*aOR* = 8.00), trust in authorities (*aOR* = 1.53), acceptability of non-pharmaceutical preventive measures (*aOR* = 2.96), and first-hand experience of COVID-19 (*aOR* = 1.32) were significant predictors of vaccine uptake after adjusting for confounding factors.

**Conclusions:**

Future strategies to promote vaccination may focus on building public trust in government and healthcare professional, and encouraging vaccinated individuals to share their vaccine uptake status via social networking.

## Highlights

• Willingness to receive a COVID-19 did not predict later vaccine uptake decision.
• Vaccine uptake by known others was associated with eightfold odds of vaccine uptake.
• Trust in authorities in combating COVID-19 predicted vaccine uptake.
• Campaigns that build public trust in government may help promote vaccination.
• Encouraging vaccinated individuals to share vaccine status may help motivate others.

## 1 Introduction

Over 5-million deaths in the world have been directly attributed to coronavirus disease 2019 (COVID-19), as well as its variants of concern (VOCs) and variants of interest (VOIs) (World Health Organization, 2021c). After the first case of COVID-19 infection being reported in December 2019, the rapid spread of infections and associated morbidity and mortality continue to bring devastating damages to all human lives in a way unmatched by any other infection in recent decades (Viswanath et al., 2021). Worldwide, public health professionals have recommended a combination of different non-pharmaceutical interventions to slow down the spread of the COVID-19. Face masks wearing, social distancing, restrictions on gathering and travelling, and curfews are commonly adopted preventive measures. Yet, it has been clear that non-pharmaceutical measures alone are not able to end the COVID-19 pandemic (Weinstraub et al., 2021). Effective and safe vaccines are therefore critical to reduce COVID-19 infections and transmissions.

As a result of the concerted global effort, the development of vaccines against COVID-19 has progressed at an unprecedented speed. According to the World Health Organization (2021b), there are 13 vaccines having completed the evaluation process, 6 under on-going evaluations, 132 in clinical development, and 194 in pre-clinical development as of November 26, 2021. The rapid development and manufacturing of these vaccines has raised public concerns over their long-term safety and efficacy. Concerns are especially great over several leading contenders developed based on new technology platforms such as genetically engineered RNA and DNA, and they may greatly influence on one's intention to receive a vaccine (Weinstraub et al., 2021).

Successful vaccinations rely on high coverage of target recipients. Engaging more eligible individuals to receive a vaccine is one of the first steps to reduce the death toll of the COVID-19 infections and bring the pandemic to an end. According to Our World in Data (Mathieu et al., 2021), almost 8-billion doses of COVID-19 vaccines have been administered worldwide, and approximately 54% of the world population has received at least the first dose of a vaccine by the end of November 2021. Vaccination coverage is generally higher among developed and high-income countries, such as United States (69%), United Kingdom (77%), Canada (79%), and Singapore (93%). In contrast, the situation in low-income countries is far from satisfactory: Coverage in some African countries is as low as 3% (Nigeria) and 6% (Ethiopia). With regard to the threats of breakthrough infections (i.e., infections of those who have been vaccinated) and reinfections with COVID-19 and its variants (D'Souza, 2021; Klompas, 2021), the previous goal of 55%–82% vaccination rate to achieve herd immunity may become insufficient. An even higher level of vaccination or the implementation of third-dose boosters may be needed (Tré-Hardy et al., 2021). Therefore, promoting vaccine uptake and ultimately increasing vaccination coverage remains as an urgent task.

In Hong Kong, the COVID-19 Vaccination Programme has officially been launched since February 26, 2021. Residents aged 12 years or above are eligible for receiving free vaccination against COVID-19. Two types of vaccines are available in Hong Kong: Comirnaty (BNT162b2 mRNA vaccine, Pfizer-BioNTech), which uses an mRNA technology platform, and CoronaVac (Sinovac), which uses an inactivated virus technology platform. As of November 1, 2021, the minimum age for receiving Comirnaty (Pfizer-BioNTech) is 12 whilst that for receiving CoronaVac (Sinovac) is 18. Eligible residents are granted choices with whether to receive vaccination and which vaccine to inoculate. Two doses of the same type of vaccine are recommended to most eligible individuals, except for those who are immunocompromised, aged 60 or above, having chronic medical conditions, healthcare workers, and workers exposed to high risks for COVID-19. For those groups of individuals, the Hong Kong government has started encouraging their uptake of a third dose of vaccine since October 27, 2021. By the end of November 2021, over 9-million doses of COVID-19 vaccines in Hong Kong and about 4.7-million people have received at least one dose of the vaccine, representing a coverage rate of 70% of the eligible population (The Government of the Hong Kong Special Administrative Region, 2021).

The emergence of the latest variant of COVID-19, Omicron (B.1.1.529), has posed new challenges to the world. First reported in South Africa in late November 2021, the Omicron variant has been regarded as a VOC because of its great number of mutations and highly infectious nature (World Health Organization, 2021a). Since its first discovery, the high transmissibility and extensive immune escape of the Omicron variant has evoked new waves of infection, leading to global concern and panic (Xu et al., 2022). Breakthrough infection has been recorded worldwide, and evidence has shown that currently approved vaccines may have compromised protection efficacy (Cele et al., 2021). Fortunately, it is suggested that current vaccines are still sufficient to protect individuals from developing severe COVID-19 (Mackay, 2021). In this sense, promoting vaccination does not only help achieve herd immunity at a community level, but also helps protect vaccinated people from severe illness at an individual level (Centers for Disease Control and Prevention, 2021).

Despite the potential benefits of vaccination, there remain a certain proportion of individuals who refuse or feel hesitate to receive a vaccine. Vaccine hesitancy refers to the reluctance or refusal to receive a vaccine despite availability of vaccination services, and has been listed by the WHO as a major threat to global health (World Health Organization, 2021d). Recent studies have shown that vaccine acceptance or hesitancy can be greatly influenced by socio-demographic factors (e.g. age, gender, ethnicity, etc.) (Aw et al., 2021; Burke et al., 2021; Kelly et al., 2021; Ogilive et al., 2021), psychosocial factors (e.g. health beliefs, perceptions, etc.) (Kelly et al., 2021; Mahmud et al., 2021; Nomura et al., 2021), disease-related factors (first-hand experience of the disease, trust in authorities in combating the disease, etc.) (Aw et al., 2021; Burke et al., 2021; Chu & Liu, 2021; Nomura et al., 2021; Soares et al., 2021), and vaccine-related factors (e.g. concerns over vaccine safety, accessibility to the vaccine, etc.) (Aw et al., 2021; Bono et al., 2021; Burke et al., 2021; Chu et al., 2021; Leng et al., 2021; Soares et al., 2021). Nonetheless, most existing research was conducted before the start of universal vaccination programmes and failed to determine predictors of actual vaccine uptake decisions.

In the battle against the COVID-19 and its variants, vaccines continue to provide irreplaceable protection at both individual and community levels. Determining predictors and correlates of the actual vaccine uptake may be an essential first step in developing effective health interventions to promote vaccination. Yet, existing COVID-19 literature mostly focused on vaccine receive intention and hesitancy, and whether the predictors of vaccine receive intention can predict actual vaccine uptake is unclear. To fill the research gap and aid strategic promotion of COVID-19 vaccination, this longitudinal study prospectively tracked individuals' willingness to vaccinate and their actual uptake decision over time, and examined potential predictors of their decisions using a large, population-based of Chinese residents in Hong Kong. Our primary research questions were (i) what factors predicted the actual vaccine uptake decision; and (ii) whether willingness to vaccinate indicated before would predict actual vaccine uptake. Based on the findings of previous studies (Aw et al., 2021; Bono et al., 2021; Burke et al., 2021; Chu et al., 2021; Kelly et al., 2021; Leng et al., 2021; Mahmud et al., 2021; Nomura et al., 2021; Ogilive et al., 2021; Soares et al., 2021), this study included potential predictors at different levels: (i) Socio-demographic factors, which included age, gender, highest education attainment, and economic status; (ii) COVID-19 related health beliefs and perceptions, which included perceived risks of contracting COVID-19, perceived benefits and obstacles, perceived social norms, acceptability of COVID-19 preventive measures by government, and trust in government and healthcare professionals in combating COVID-19; (iii) COVID-19 related experiences, which included individuals’ first-hand experience of COVID-19 and previous experience of other pandemic or epidemic (e.g., SARS, MERS, etc.); and (iv) vaccine related perceptions, which included willingness to receive a COVID-19 vaccine, concerns about vaccine safety, and vaccine uptake status by known others. To the best of our knowledge, this study was the first to compare and explore the association between willingness to accept a COVID-19 vaccine indicated before the start of the public vaccination programme and vaccine uptake decision at a later stage. It was also one of the first to systematically investigate the predictors of actual vaccine uptake, instead of vaccine receive intention, acceptance, or hesitancy, at different levels.

## 2 Materials and methods

### 2.1 Study setting and design

A population-based, longitudinal prospective survey study was conducted among the general population Hong Kong. Surveys were administered to a randomly drawn sample through telephone calls at two time points: T1 baseline, (December 2020–January 2021, before the implementation of the Vaccination Programme) and T2 follow-up (June 2021–July 2021, about 4–5 months after the start of the Vaccination Programme).

### 2.2 Participants and data collection

All Hong Kong residents aged 18 or above who were able to communicate in Cantonese or Mandarin were eligible to participate in this study. Data were collected via telephone surveys by a team of trained research assistants with the use of structured questionnaires. Telephone numbers were selected randomly using a multi-stage procedure that has been applied in many population-based telephone surveys in Hong Kong (Wu et al., 2019; Zhao et al., 2019). Random numbers were drawn from a local directory that covered both landline and mobile numbers in Hong Kong. Numbers drawn were then used as “seeds” to generate another set of numbers with the “last digit plus/minus one/two” method. Duplicated numbers were filtered, and remaining numbers were mixed in random order to give the final set of 6,000 telephone numbers.

Trained research assistants contacted eligible participants by telephone calls from 9am to 9pm on weekdays under the supervision of the research team. A maximum of five attempts of contact were made for each telephone number before it was classified as “non-contact”. For landline numbers, if there were more than one eligible individual in the household, participants were selected with the last birthday method. Participants were explained about the study objectives and their rights to withdraw from the study or omit any question. Oral consent was sought before the survey began. Both of the T1 baseline and T2 follow-up surveys comprised 50–60 questions that took approximately 20 min to complete. All data were collected using a computer-assisted telephone interview (CATI) system, which facilitated real-time data input and consolidation. Participants were asked to provide their preferred contact number for T2 follow-up after the completion of T1 survey. Ethical approval was obtained from the institutional review board of the Hong Kong Polytechnic University before the start of this study. All identifying information was removed in the dataset and all data were analysed in aggregate to ensure confidentiality and anonymity.

### 2.3 Measures

#### 2.3.1 Outcome variable

*Vaccine uptake status*. An item, (“Have you received at least the first dose of COVID-19 vaccine?), was used to record the vaccine uptake status of the participants at T2. As the Vaccination Programme had not been launched at T1, we did not assess vaccine uptake status in our baseline surveys. Responses were captured in a dichotomous “yes/no” basis.

#### 2.3.2 Independent variables (T1 baseline)

##### 2.3.2.1 COVID-19 related experiences, health beliefs and perceptions

*Perceived risks* was measured with the item (“It is likely for me to contract COVID-19.”), which probed a dichotomous “yes/no” answer. *Perceived benefits* and *perceived obstacles* of the COVID-19 preventive measures initiated by the government were assessed with three and six items, respectively. Sample items included (Perceived benefits: “I believe the COVID-19 preventive measures are effective in controlling the spread of COVID-19.”) and (Perceived obstacles: “COVID-19 preventive measures are annoying and time-wasting.”). *Perceived social norms* were measured with seven items, including (“Most people I know comply to the preventive measures.”) and (“My family and friends think that I should comply to the preventive measures.”). *Acceptability of the preventive measures* were captured with six items, with each assessing the level of acceptance to one of the six preventive measures: compulsory mask wearing, restrictions on dine-in services, restrictions on group gatherings, compulsory testing of COVID-19 among specific high-risk groups, compulsory quarantine of individuals arriving Hong Kong, and compulsory quarantine of specific groups of citizens when in need. All items assessing perceived benefits, perceived obstacles, perceived social norms, and acceptability were rated upon a 5-point Likert scale, from 1 (“Strongly disagree”) to 5 (“Strongly agree’). Item score were averaged to give a scale score. Higher mean scores indicated higher levels of the specific health beliefs and perceptions. In this study, internal consistencies of the scales were good, with Cronbach's alpha ranging from 0.78 (perceived benefits) to 0.92 (perceived social norms).

##### 2.3.2.2 Vaccine related perceptions

Participants rated their *willingness to receive a vaccine* at T1 with the item (“I will receive the COVID-19 vaccines when they are available.”) against a 5-point Likert scale with responses ranged from 1 (“Strongly disagree”) to 5 (“Strongly agree”). *Concerns about vaccine safety* were assessed with a single item (“I am concerned about the safety and side effects of COVID-19 vaccines.”), which was rated against a 5-point scale ranging from 1 (“No/Little concerns”) to 5 (“Great concerns”).

#### 2.3.3 Independent variables (T2 follow-up)

##### 2.3.3.1 Experiences related to COVID-19 and other epidemics or pandemics

Participants reported whether people around them had contracted or been suspected to contract COVID-19 with three items, which covered participants’ family, friends, and neighbours. They also reported whether they or their family had ever contracted SARS, avian flu (H5N1 and its subtypes), or swine flu (H1N1) before. All items were rated on a 5-point Likert scale, from 1 (“None of them”) to (“Almost all of them”). Item scores were averaged to give mean scores for the variables *current experiences of COVID-19* and *previous experiences of other pandemics and epidemics*.

##### 2.3.3.2 COVID-19 related health beliefs and perceptions

Participants rated their levels of *trust in the Hong Kong government and healthcare professionals* in controlling the spread of COVID-19 with two items. Each item was rated on a 11-point Likert scale, from 0 (reflecting lowest level of trust) to 10 (reflecting highest level of trust).

##### 2.3.3.3 Vaccine related perceptions

*Vaccine uptake by others* was measured with three items, including (“My colleagues/family or relatives/friends have received vaccines.”). All items were rated on a 5-point Likert scale, from 1 (“None of them”) to (“Almost all of them”). Item scores were averaged to give a mean scale score, with a higher score indicating more people around the participants have received the COVID-19 vaccines.

#### 2.3.4 Covariates

Demographic factors of the participants, including *gender*, *age*, *highest education attainment*, and *economic activity status* were measured as covariates in the study.

### 2.4 Statistical analysis

Analyses were performed using SPSS 26.0, and p-values smaller than 0.05 were deemed statistically significant in this study.

To ensure the representativeness of the study findings, raw data were first weighted according to the latest sex-age distribution in the Hong Kong population provided by the Census and Statistics Department (2021). Descriptive statistical analyses were performed among all variables. Demographic variables were compared for gender differences. Mean scores or proportions of all variables were computed and compared between vaccinated participants and unvaccinated participants.

To examine the predictors and correlates of the COVID-19 vaccine uptake, a hierarchical logistic regression analysis was conducted. Hierarchical regression analysis is a commonly used way to show if variables of interest can explain a statistically significant amount of variance in the dependent variable after adjusting for all other variables, and is a framework which helps model comparison. In this study, the dependent variable was the vaccine uptake status (1 “yes”/0 “no”). The four blocks of independent variables included: (i) demographic background (i.e., gender age, education attainment, and economic activity status); (ii) perceived risks; (iii) COVID-19 related experiences, health beliefs, and perceptions (including current experiences of COVID-19; previous experiences of other pandemics and epidemics; perceived benefits, perceived obstacles, perceived social norms, and acceptability of COVID-19 preventive measures; and trust in authorities); and (iv) vaccine related perceptions (i.e., willingness to receive a vaccine, concerns about vaccine safety, and vaccine uptake by others). In our analysis, each block of variables was entered to a previous model at each step, so that later models would include the models in previous steps. Odds ratios of our dependent variable were adjusted with variables in previous blocks and other variables in the same block of the regression model. Multicollinearity was checked before performing the regression analyses.

Hierarchical regression was regarded as a more appropriate tool than the ordinary stepwise logistic regression in this study as the predictor variables were very possibly correlated with each other. For example, health belief model factors which were entered in block 2 and block 3 of independent variables (including perceived risks, perceived benefits, perceived obstacles, etc.) were often demonstrated to be associated with one's willingness to receive a vaccine, which was entered in block 4 of independent variables (Detoc et al., 2020; Wong et al., 2021). The correlations among predictor variables may lead to confounding effects that reduce the generalisability of the findings. The use of hierarchical regression might help minimise the issue by investigating the relationships within and between different hierarchical levels of data.

## 3 Results

### 3.1 Participants’ characteristics

Among the 6,000 telephone numbers sampled, 2,784 were valid and in-use. A total of 1,255 participants completed the telephone survey at T1 (response rate = 45.1%), and 1,003 of them were successfully contacted and surveyed at T2 (retention rate = 79.9%). Reasons for excluded cases at T1 included non-contact (34.8%), refusal of participation (19.4%), and language and communication barriers (0.8%).

Both unweighted and weighted data on demographic background of the participants were summarised in Table 1. Weighted data showed that the final sample comprised 47.0% of men and 54.0% of women. The modal age group was 55 years or above (41.3%), followed by 35–54 years (35.0%) and 18–34 years (23.7%). A majority of the participants received upper secondary education or above (70.0%). Almost two thirds (64.6%) were economically active at T1.

**Table 1:** Demographic characteristics of the sample (N = 1,003, weighted N = 6,134,040).

Variable	Total		By gender				Chi-square	*p*-value
			Male (n = 494, weighted n = 2881760)		Female (n = 509, weighted n = 3252280)			
	Unweighted n (%)	Weighted n (%)	Unweighted n (%)	Weighted n (%)	Unweighted n (%)	Weighted n (%)		
**Age (years)**							0.19	0.908
18–34	230 (22.9)	1453440 (23.7)	116 (23.5)	716160 (24.9)	114 (22.4)	737280 (22.7)		
35–54	329 (32.8)	2147800 (35.0)	162 (32.8)	960500 (33.3)	167 (32.8)	1187300 (36.5)		
55 or above	444 (44.3)	2532800 (41.3)	216 (43.7)	1205100 (41.8)	228 (44.8)	1327700 (40.8)		
**Education attainment**							3.36	0.500
Primary/Lower secondary	314 (31.3)	1839573 (30.0)	149 (30.2)	828481 (28.8)	165 (32.4)	1011093 (31.1)		
Upper secondary	445 (44.4)	2763422 (45.1)	221 (44.7)	1317409 (45.7)	224 (44.0)	1446013 (44.5)		
Diploma/University degree/Postgraduate degree	244 (24.3)	1531045 (24.9)	124 (25.1)	725870 (25.5)	120 (23.6)	795174 (24.4)		
**Economic activity status**							150.77	<0.001
Active	633 (63.1)	3959548 (64.6)	278 (56.3)	2163252 (75.1)	271 (53.2)	1796296 (55.2)		
Not currently active								
Student	50 (5.0)	290821 (4.7)	16 (3.2)	134061 (4.7)	25 (4.9)	156760 (4.8)		
Homemaker	147(14.7)	874824 (14.3)	61 (12.3)	22266 (0.8)	143 (28.1)	852558 (26.2)		
Retired	148 (14.8)	842317 (13.7)	148 (30.0)	473708 (16.4)	59 (11.6)	368609 (11.3)		
Unemployed/Between jobs	25 (2.5)	166529 (2.7)	24 (4.8)	88473 (3.1)	11 (2.2)	78056 (2.4)		

*Note.* Data were weighted according to the latest sex-age distribution in the Hong Kong population provided by the Census and Statistics Department (2021).

Gender difference was only observed in economic activity status (χ^2^ = 150.77, *p* < 0.001). Men were more likely to be working and economically active than women (75.1% versus 55.2%), whilst a greater proportion of women than men were homemakers and did not earn a living (26.2% versus 0.8%).

### 3.2 Vaccine uptake

Overall, 238 participants (23.7%) had received at least the first dose of COVID-19 vaccines at T2. Among all vaccinated participants, 50.9% were men, with a mean age of 50.65 years. *T*-tests revealed a significant difference in age between vaccinated participants and unvaccinated participants. The former group was significantly older than the latter (*t* = −2.13, *p* < 0.05). Details of the comparisons are presented in Table 2.

**Table 2:** Mean scores or proportions of the variables, by participants’ vaccination status at the follow-up survey (T2) (N = 1,003).

Variable	Time point	No. of items	Score range	Overall (N = 1,003)	Vaccine uptake status at T2		*t*/Chi-square	*p*-value
					Vaccinated (n = 238)	Unvaccinated (n = 765)		
**Gender**	T1	1	N/A				0.31	0.576
Male				49.3%	50.9%	48.8%		
Female				50.7%	49.1%	51.2%		
**Age**	T1	1	N/A	48.94	50.6%	48.41	−2.13	0.033
**Education attainment**	T1	1	N/A				0.99	0.609
Primary/Lower secondary				31.3%	29.1%	32.0%		
Upper secondary				44.4%	47.0%	43.6%		
Diploma/University degree/Postgraduate degree				24.3%	23.9%	24.4%		
**Economic status (active)**	T1	1	N/A	59.0%	63.4%	58.0%	1.43	0.231
**Experiences of pandemic or epidemic**								
Current experience of COVID-19	T2	3	1–5	2.24	2.33	2.22	−1.33	0.183
Previous experience of other pandemic or epidemic (e.g. SARS)	T2	3	1–5	1.69	1.69	1.69	−0.07	0.947
**COVID-19 related health beliefs and perceptions**								
Perceived risks (Risky)	T1	1	0/1	21.3%	28.6%	19.1%	9.68	0.002
Measured benefits of COVID-19 preventive measures	T1	3	1–5	4.24	4.40	4.20	−4.20	<0.001
Measured obstacles of COVID-19 preventive measures	T1	6	1–5	2.58	2.41	2.63	2.57	0.011
Acceptability of COVID-19 preventive measures	T1	6	1–5	4.40	4.63	4.33	−7.10	<0.001
Perceived social norms	T1	7	1–5	4.08	4.25	4.03	−5.10	<0.001
Trust in authority	T2	2	0–10	4.18	5.33	3.83	−11.84	<0.001
**Vaccine related perceptions**								
Willingness to receive a vaccine	T1	1	1–5	3.27	3.43	3.22	−2.43	0.015
Concerns about vaccine safety	T1	1	1–5	3.89	3.99	3.86	−2.11	0.035
Uptake by others (e.g. family, friends, colleagues, etc.)	T2	3	1–5	2.24	2.77	2.07	−15.53	<0.001

### 3.3 COVID-19 and vaccine related factors

Table 2 also shows the mean scores and proportions of other study variables. Significant differences between vaccinated participants and unvaccinated participants were found in all COVID-19 related health beliefs and perceptions. When compared with unvaccinated individuals, vaccinated participants perceived greater risks of contracting COVID-19 at T1 (19.1% versus 28.6%, χ^2^ = 9.68, *p* < 0.01). Vaccinated participants reported greater benefits, fewer obstacles, higher acceptability, and greater social norms related to the compliance to COVID-19 preventive measures; while at the same time showing greater trusts in authorities in controlling the spread of COVID-19 (all *p* < 0.05).

Significant between-group differences were also observed in all vaccine related variables. Vaccinated participants indicated greater willingness to receive COVID-19 vaccines at T1 (mean score: 3.43 versus 3.22, *t* = −2.43, p < 0.05), and had more people around having received the vaccines (mean score: 2.77 versus 2.07, *t* = −15.53, p < 0.001). Surprisingly, vaccinated participants reported greater concerns about the safety and side effects of the vaccines at T1 (mean score: 3.99 versus 3.86, *t* = −2.11, p < 0.05).

### 3.4 Predictors and correlates of COVID-19 vaccine uptake

Findings from the hierarchical regression analysis are presented in Table 3. In model 2, perceived risks of contracting COVID-19 at T1 were a significant predictor of vaccine uptake at T2 (adjusted odds ratio (*aOR*) = 1.62 [95% confidence interval (CI) = 1.15–2.28], *p* < 0.01). However, when COVID-19 related experiences, health beliefs, and perceptions variables were added in model 3, the effect of perceived risks became non-significant (*aOR* = 0.79 [0.50–1.23], *p* > 0.05). In the expanded model, participants’ acceptability of COVID-19 preventive measures at T1 and trusts in authorities at T2 were two significant predictors of vaccine uptake. A greater acceptability of preventive measures (*aOR* = 2.28 [1.53–3.40], *p* < 0.001) and a greater trust in authorities in controlling COVID-19 (*aOR* = 1.76 [1.56–1.97], *p* < 0.001) predicted higher odds of the vaccine uptake after the adjustment for confounding variables.

**Table 3:** Hierarchical regression model of factors associated with vaccination status (N = 1,003).

Variable	Vaccine uptake			
	Model 1	Model 2	Model 3	Model 4
	OR (95% CI)	OR (95% CI)	OR (95% CI)	OR (95% CI)
**Demographic factors**				
Gender (ref. Female)				
Male	1.03 (0.76–1.39)	1.02 (0.75–1.38)	0.99 (0.70–1.40)	0.98 (0.66–1.44)
Age	1.02** (1.01–1.03)	1.02** (1.00–1.03)	1.00 (0.99–1.01)	1.01 (0.99–1.02)
Education (ref. Diploma/University degree/Postgraduate degree)				
Primary/Lower secondary	0.63 (0.38–1.06)	0.65 (0.39–1.09)	0.84 (0.48–1.48)	0.86 (0.45–1.65)
Upper secondary	0.89 (0.60–1.32)	0.91 (0.61–1.35)	1.01 (0.65–1.56)	1.09 (0.66–1.80)
Economic activity (ref. Inactive)				
Active	1.32 (0.93–1.88)	1.34 (0.94–1.91)	1.40 (0.95–2.07)	1.17 (0.76–1.80)
**Perceived risks**				
Risky (ref. not risky)		1.62** (1.15–2.28)	0.79 (0.50–1.23)	0.78 (0.47–1.28)
**COVID-19 related experiences, health beliefs, and perceptions**				
Current experience of COVID-19			1.06 (0.86–1.30)	1.32* (1.04–1.69)
Previous experience of other pandemic or epidemic			1.07 (0.85–1.33)	1.00 (0.78–1.29)
Perceived benefits of COVID-19 preventive measures at T1			0.85 (0.60–1.23)	0.84 (0.56–1.25)
Perceived obstacles of COVID-19 preventive measures at T1			1.05 (0.84–1.32)	0.94 (0.73–1.21))
Acceptability of COVID-19 preventive measures at T1			2.28*** (1.53–3.40)	2.96*** (1.89–4.63)
Perceived social norms at T1			1.12 (0.74–1.70)	1.27 (0.81–1.99)
Trust in authority at T2			1.76*** (1.56–1.97)	1.53*** (1.34–1.75)
**Vaccine related perceptions**				
Willingness to receive a vaccine at T1				0.91 (0.75–1.11)
Concerns about vaccine safety at T1				1.25 (0.99–1.58)
Uptake by others (e.g. family, friends, colleagues, etc.)				8.00*** (5.59–11.45)
**Model statistics**				
Cox and Snell *R*^2^	0.012	0.019	0.158	0.296
Nagelkerke *R*^2^	0.018	0.028	0.239	0.447

Note. *p < 0.05. **p < 0.01. ***p < 0.001.

When vaccine related perceptions were added in the final model, current experiences of COVID-19 (*aOR* = 1.32 [1.04–1.69], *p* < 0.05) and vaccine uptake by known others (*aOR* = 8.00 [5.59–11.45], *p* < 0.001) were found to be significantly associated with vaccine uptake in addition to the significant effects of acceptability of preventive measures (*aOR* = 2.96 [1.89–4.63], *p* < 0.001) and trust in authorities (*aOR* = 1.53 [1.34–1.75], *p* < 0.001). When controlling for other possible confounding factors, higher odds of COVID-19 vaccine uptake could be associated with more people around having been contracted with COVID-19, more people around having received the vaccines, greater acceptability of COVID-19 preventive measures implemented by the government, and trust in authorities in controlling the spread of COVID-19.

## 4 Discussion

### 4.1 Vaccine uptake

Understanding the determinants of vaccine uptake is crucial during a pandemic. This longitudinal study tracked prospectively individuals’ willingness to receive a vaccine right before COVID-19 vaccines were available and their actual vaccine uptake status about 4–5 months after the start of the public vaccination programme, as well as to investigate the predictors of actual vaccine uptake.

By the end of July 2021, approximately one quarter of the study sample (>18 years old) reported that they had received at least one dose of the COVID-19 vaccines, a figure which is lower than the official statistics that reported a vaccine uptake rate of 48% among residents aged 12 or above (as of July 31, 2021; World Health Organization, 2021a). At the same period of time, the global vaccination rates ranged from 11% (Africa) to 72% (North America) (Mathieu et al., 2021). Comparatively, the vaccine uptake rate in Hong Kong by July 2021 was lower than the average rates in Asia (62%) and among high-income countries (>50%–90%) (Mathieu et al., 2021; Rouw et al., 2021), reflecting that individuals in Hong Kong might have greater vaccine hesitancy than their Asian or high-income counterparts. To achieve herd immunity, researchers have previously suggested at least 60% coverage of a vaccine (Anderson et al., 2020). Yet, recent findings showed that breakthrough infections and reinfections might not be a rare phenomenon (D'Souza et al., 2021). This might indicate our urgent need of higher vaccination coverage in the world in order to achieve herd immunity and life-long protection.

### 4.2 Predictors of vaccine uptake

Surprisingly, this study demonstrated that previous intention to receive a vaccine against COVID-19 might not necessarily be associated with the decision of vaccine uptake. Although our data showed that previous level of willingness to vaccinate was significantly greater among vaccinated than unvaccinated, such level of willingness did not significantly predict actual vaccine uptake status when other factors were adjusted for. In this study, some individuals might have changed their intention to receive the COVID-19 vaccines or decided to delay their vaccination after the implementation of the public vaccination programme. The change in willingness to vaccinate among the population has been indicated in a repeated cross-sectional study using a sample of 2,047 working people in Hong Kong (Wang et al., 2021). From February to August 2020, there was a general decline from 44% to 35% in the acceptance rate of COVID-19 vaccines, accompanied by an increase in concerns over vaccine safety across time. Similarly in a survey study in China, over half of the respondents who stated an intention to vaccinate would choose to delay the vaccination until vaccines against COVID-19 were confirmed safe (Wang et al., 2020).

A possible reason behind the change of vaccine uptake intention among individuals in Hong Kong may be the shift of attention from the risks of disease to the risks of vaccine when the disease becomes less severe (Dubé et al., 2013). Indeed, it has been argued that, “vaccination is victim of its own success (p.1767).” When vaccination programmes are successful in preventing the spread of the diseases, the risks of contracting the specific diseases may become less visible to the public (Schwarz & Caplan, 2011). Attention may, instead, be directed to the safety issues of the vaccination programme. Hoping to end the spread of COVID-19 which has brought devastating impacts on every aspect in our lives, the development, testing, and manufacturing of vaccines against COVID-19 has been progressed faster than that of any other vaccine in history (Mathieu et al., 2021). One of the drawbacks of rapid development of vaccine might be the unpredicted long-term efficacy, safety, and side effects, especially when several major vaccines are based on the new mRNA and DNA platforms that lack track records (Weinstraub et al., 2021). During the time when T2 surveys in this study were conducted, the risks of COVID-19 in Hong Kong were relatively low, as almost no new local cases had been reported in the city for several months (The Government of the Hong Kong Special Administrative Region, 2022). It could be expected that perceived risks of COVID-19 among the public during T2 would be lower than those during T1, when Hong Kong was under the fourth epidemic wave of COVID-19 outbreak. When perceived risks of COVID-19 became lower, vaccine safety and effectiveness might place stronger influence on vaccine uptake (Bono et al., 2021; Chu et al., 2021; Dula et al., 2021; Nomura et al., 2021). The shift of attention might inevitably lower the public's intention to receive the vaccines with unknown long-term safety issues, leading to the non-significant predicting power of intention at T1 on actual decision at T2. To encourage individuals to receive a vaccine, especially during the time when the risks of the disease is not severe, it is therefore of vital importance to deliver effective education to the general public in order to minimise misinformation and unnecessary concerns over the vaccine safety.

In this study, one of the strongest predictors was vaccine uptake by known individuals (e.g., family, friends, colleagues, etc.). The likelihood of receiving COVID-19 vaccines could be eightfold among those who indicated known individuals had received the vaccine. This finding is in line with past research on other vaccinations that widespread vaccine uptake could induce others to receive the vaccine through the process of herding (Broniatowski et al., 2018). Herding is one of the social factors that have been shown to dominate one's free-riding decision in vaccine uptake (Agranov et al., 2021). In the case of COVID-19 vaccination, individuals might follow the crowd (in particular people around) to receive the vaccine as they were convinced by others' action that vaccines were safe. Uptake by people around might also create social pressure that promoting altruistic behaviours while reducing free-riding motives (Leng et al., 2021), which further motivated individuals to receive the vaccine (Agranov et al., 2021). In fact, some preliminary research on COVID-19 vaccine acceptance has reported that vaccine uptake by others could be significantly associated with intentions to receive a vaccine (Burke et al., 2021; Leng et al., 2021; Mahmud et al., 2021; Nomura et al., 2021). Our findings further strengthened the evidence supporting the predicting power of uptake by others on people's vaccine uptake decision. As another social factor that greatly affect vaccine uptake in past research (Agranov et al., 2021; Latkin et al., 2021), perceived social norm before the start of the vaccination programme did not play a significant role in predicting uptake decision, although the level of perceived social norm favouring vaccination was higher among vaccinated than unvaccinated. Despite the possibility of an actual non-significant association between social norm and vaccine uptake, the limited role played by social norm might be due to the dynamic change of it across time that could not be measured in this study. Perceived social norm was only measured at T1 (before the commencement of the vaccination programme). Individuals might have changed their perceived norms over time, and vaccine uptake decision might be more strongly associated with recent perceptions than previous perceptions.

Trust in authorities, the most commonly stated correlate of acceptance and intention to receive a vaccine (Aw et al., 2021; Burke et al., 2021; Latkin et al., 2021; Lazarus et al., 2021; Leng et al., 2021; Nomura et al., 2021), was demonstrated as a significant predictor in vaccine uptake decision in this study. The strong association between trust and vaccine uptake highlights the importance of assurances from authorities in promoting public confidence in vaccines, which could serve to motivate vaccine uptake by reducing fears or worries related to vaccine safety (van der Weerd et al., 2011; Viswanath et al., 2021). In this study, it is noteworthy that the overall levels of trust in authorities were low, and the low mean score reflected a general distrust in the authorities, especially the government, in handling COVID-19. The government has been criticised for acting for political motives instead of for citizens’ interests (Chan, 2021). In order to boost vaccine uptake in the future, the Hong Kong government may consider increasing the transparency and public participation when making decisions related to combating COVID-19.

Current experience of COVID-19 (e.g., whether individuals had family or friends contracted COVID-19) and level of acceptability of governmental COVID-19 preventive measures were the other two significant predictors of vaccine uptake. First-hand experience with the disease is believed to heighten fears related to the contraction of COVID-19, which might then counter the negative effects of over-optimism on vaccine uptake decision (Chu & Liu, 2021). Our findings provide additional evidence for the potential effectiveness of stressing personal relevance of COVID-19 in promoting vaccine uptake. The significant association between acceptability of governmental preventive measures and vaccine uptake echoes the findings in a recent research in Portugal, that individuals who expressed negative feelings on government measures would show lower vaccine hesitancy (Soares et al., 2021). It could be possible that both acceptance to vaccination and acceptance to non-pharmaceutical governmental preventive measures were rooted from a high level of trust in authorities (Nivette et al., 2021).

Unlike other studies on the effects of socio-demographic background and health beliefs on intention to vaccinate (Aw et al., 2021; Chu et al., 2021; Kelly et al., 2021; Nomura et al., 2021; Soares et al., 2021), this study found that none of the socio-demographic factors (e.g., age and gender) or Health Belief Model factors (e.g., perceived risks and perceived benefits) was predictive of vaccine uptake status after the adjustment for other variables. These findings may shed lights on the design of effective vaccination promotion campaign in Hong Kong. Instead of focusing on the risks, benefits, and barriers of receiving a COVID-19 vaccine, the government may put greater efforts in building trust with the public, and relate individuals’ first-hand experience of COVID-19 to vaccination, and emphasise the widespread uptake of vaccines in the city.

### 4.3 Implications

With the emergence of the COVID-19 pandemic, the rapid spread of infection over the world, and the great harms it has brought to human lives, the need for promoting vaccine uptake to achieve herd immunity and to protect individuals against serious illness and hospitalisation is urgent. However, many individuals remain doubtful to the long-term efficacy and safety of the vaccines against COVID-19, and show hesitancy or even refusal to vaccination.

Findings in this study provide insights on the development of evidence-based campaigns to promote vaccine uptake. Indeed, public awareness programmes tailored to specific needs have been found effective in increasing vaccine uptake rates in other infections (McAteer et al., 2020). While disseminating information about vaccine safety and efficacy may be useful, they are demonstrated to be insufficient to affect individuals' vaccine uptake decision. The strong predictive power of vaccine uptake by known others warrants the use of strategies that promote social norms. Peers can be important sources of information regarding COVID-19 vaccination. Getting to know that family members, friends, or other acquaintances have received a vaccine without developing serious side effects may lower one's concern about vaccine safety issues, one of the major obstacles to vaccine acceptance. In view of this, social network diffusion can be one of the promising ways to promote vaccination. Campaigns that facilitate peer networks for vaccinated individuals to share their vaccine uptake status on social network may increase the opportunity to induce their acquaintances to follow via herding behaviours and social pressure.

Concerning the association between trust in authorities and vaccine uptake, it is strongly recommended that government officials and healthcare professionals should exert more effort in promoting a trustable and sincere image, and enhancing the transparency of information relevant to vaccines against COVID-19. According to a previous research on other public health programmes, healthcare professionals should no longer assume that the public would simply trust them because of their social status or expertise (Ward, 2017). Building trust in authorities may increase public confidence and reduce scepticism to the vaccination programme, which may help optimise vaccine uptake and coverage. To enhance public trust in the authorities, it has been suggested that governments should take the lead to partner and support relevant departments and organisations to conduct well-managed engagement of the community (The Organisation for Economic Co-operation and Development, 2021). Governments should ensure transparent actions, timely information release, and coherent communications with public to address the possible misinformation about vaccines, and should manage public expectations especially when new challenges of the pandemic emerge.

### 4.4 Strengths and limitations

This study was among the first to explore the predictors of the uptake of vaccines against COVID-19 using a population-based, random sample. The longitudinal design allowed us to track the same group of individuals on their willingness to receive a vaccine as indicated before the implementation of the vaccination programme and their actual uptake decision. The non-significant association between previous willingness to vaccinate and actual uptake decision provides important insights on the development of effective campaign to maximise the reception of vaccines. Components that could increase acceptance or intention to receive a vaccine might not necessarily be effective in promoting vaccine uptake. Future research may explore more on the determinants of vaccine uptake using more recent data.

Although this study has unique strengths that helped extend current knowledge, several inevitable limitations might reduce the generalisability of the current findings. Due to the use of telephone survey, there might be an uneven distribution of the participants. Our sample might be suffered from sampling biases and deviated from the population. For example, individuals without a mobile phone or landline phone were excluded in the selection process. To minimise the impact of the potential biases, weights were applied to the data. Also, it should be noted that our exploration of socio-demographic predictors was not exhaustive. Some factors that were found correlated with vaccination intention (such as income, occupation, and ethnicity) were not included. As this study employed telephone surveys, it was important to keep the survey concise so as to reduce dropouts. Future research may consider using other methods of data collection, such as online surveys and face-to-face interviews, and include more items to get a better understanding of the predictors of vaccine uptake. Furthermore, the variables grouping and their order of entry had no absolute answer. It is always possible for alternative ways of grouping and entry order. Although we have developed the model based on previous research evidence, there could still be errors in the model that might lead to a reduced generalisability of the findings. Finally, this study did not assess the workplace or school requirements of vaccination as well as the need to fulfil dine-in or gathering vaccine bubbles requirements by the government among the participants. Future research may explore these factors in predicting vaccine uptake and how they could be utilised to increase vaccination rates.

## 5 Conclusions

The COVID-19 pandemic remains as a global crisis with tremendous economic and social costs. As non-pharmaceutical preventive measures such as mask wearing and social distancing alone can no longer stop the spread of the infection, the implementation of effective vaccination may emerge as the essential step towards curtailing the spread of COVID-19. A successful vaccination programme relies on high coverage of target recipients. This study demonstrates that vaccine uptake decision-making can be a dynamic process, and may not necessarily be associated with previous willingness to receive a vaccine. This highlights the urgent need for on-going research to explore the most up-to-date profiles concerning determinants of vaccine uptake in order to inform more evidence-based campaign to promote vaccination in the future. Based on the findings of this study, it is recommended to develop a promotion campaign that aims at building public trust in the government and healthcare professionals, relating the first-hand experience of COVID-19 among individuals to vaccination, and engaging social network to encourage sharing of vaccine uptake status to induce more individuals to receive a COVID-19 vaccine.

## Ethics

This study has been approved by the Human Research Ethics Committee at the Hong Kong Polytechnic University (HSEARS20200814002).

## Funding

This project is funded by the Hong Kong Food and Health Bureau, Grant Number: COVID190216.

## Author statement

Elsie Yan: Conceptualization; Formal analysis; Funding acquisition; Methodology; Project administration; Supervision; Writing - original draft; Writing - review & editing.

Daniel W.L. Lai: Conceptualization; Funding acquisition; Supervision; Writing - review & editing.

Haze K.L. Ng: Conceptualization; Formal analysis; Project administration; Writing - original draft; Writing - review & editing.

Vincent W.P. Lee: Conceptualization; Funding acquisition; Project administration.

## References

[bib1] Agranov M., Elliot M., Ortoleva P. (2021). The importance of social norms against strategic effects: The case of COVID-19 vaccine uptake. Economics Letters.

[bib2] Anderson R.M., Vegvari C., Truscott J., Collyer B.S. (2020). Challenges in creating herd immunity to SARS-CoV-2 infection by mass vaccination. Lancet.

[bib3] Aw J., Seng J.J.B., Seah S.S.Y., Low L.L. (2021). COVID-19 vaccine hesitancy: A scoping review of literature in high-income countries. Vaccines.

[bib4] Bono S.A., Villela E.F.M., Siau C.S., Chen W.S., Pengpid S., Hasan T., Sessou P., Ditekemena J.D., Amodan B.O., Hosseinipour M.C., Dolo H., Fodjo J.N.S., Low W.Y., Colebunders R. (2021). Factors affecting COVID-19 vaccine acceptance: An international survey among low- and middle-income countries. Vaccines.

[bib5] Broniatowski D.A., Jamison A.M., Qi S.H., Alkulab L., Chen T., Benton A., Quinn S.C., Dredze M. (2018). Weaponized health communication: Twitter bots and Russian trolls amplify the vaccine debate. American Journal of Public Health.

[bib6] Burke P.F., Masters D., Massey G. (2021). Enablers and barriers to COVID-19 vaccine uptake: An international study of perceptions and intentions. Vaccine.

[bib7] Cele S., Jackson L., Khan K., Khoury D., Moyo-Gewte T., Moosa M.S., Davenport M., de Oliveira T., Moore P.L.A., Sigal A. (2021). https://www.medrxiv.org/content/10.1101/2021.12.08.21267417v3.

[bib8] Census and Statistics Department (2021). https://www.censtatd.gov.hk/en/web_table.html?id=1A.

[bib9] Centers for Disease Control and Prevention (2021). Benefits of getting a COVID-19 vaccine. https://www.cdc.gov/coronavirus/2019-ncov/vaccines/vaccine-benefits.html.

[bib10] Chan R.K.H. (2021). Tackling COVID-19 risk in Hong Kong: Examining distrust, compliance and risk management. Current Sociology.

[bib11] Chu H., Liu S. (2021). Integrating health behaviour theories to predict American's intention to receive a COVID-19 vaccine. Patient Education and Counseling.

[bib12] Detoc M., Bruel S., Frappe P., Tardy B., Botelho-Nevers E., Gagneux-Brunon A. (2020). Intention to participate in a COVID-19 vaccine clinical trial and to get vaccinated against COVID-19 in France during the pandemic. Vaccine.

[bib13] D'Souza G. (2021). https://publichealth.jhu.edu/2021/what-is-herd-immunity-and-how-can-we-achieve-it-with-covid-19.

[bib14] Dubé E., Laberge C., Guay M., Bramadat P., Roy R., Bettinger J.A. (2013). Vaccine hesitancy: An overview. Human Vaccines & Immunotherapeutics.

[bib15] Dula J., Mulhanga A., Nhanombe A., Cumbi L., Júnior A., Gwasvaira J., Fodjo J.N.S., Villela E.F.M., Chicumbe S., Colebunders R. (2021). COVID-19 vaccine acceptability and its determinants in Mozambique: An online survey. Vaccines.

[bib16] Kelly B.J., Southwell B.G., McCormack L.A., Bann C.M., MacDonald P.D.M., Frasier A.M., Bevc C.A., Brewer N.T., Squiers L.B. (2021). Predictors of willingness to get a COVID-19 vaccine in the U.S. BMC Infectious Diseases.

[bib17] Klompas M. (2021). Understanding breakthrough infections following mRNA SARS-CoV-2 vaccination. JAMA.

[bib18] Latkin C., Dayton L.A., Yi G. (2021). COVID-19 vaccine intentions in the United States, a socio-ecological framework. Vaccine.

[bib19] Lazarus J.V., Ratzan S.C., Palayew A., Gostin L.O., Larson H.J., Rabin K., Kimball S., El-Mohandes A. (2021). A global survey of potential acceptance of a COVID-19 vaccine. Nature Medicine.

[bib20] Leng A., Maitland E., Wang S., Nicholas S., Liu R., Wang J. (2021). Individual preferences for COVID-19 vaccination in China. Vaccine.

[bib22] Mackay H. (2021). https://www.bbc.com/news/uk-59442141.

[bib23] Mahmud I., Kabir R., Rahman M.A., Alradie-Mohamed A., Vinnakota D., Al-Mohaimeed A. (2021). The Health Belief Model predicts intention to receive the COVID-19 vaccine in Saudi Arabia: Results from a cross-sectional survey. Vaccines.

[bib24] Mathieu E., Ritchie H., Ortiz-Ospina E., Roser M., Hasell J., Giattino C., Rodes-Guirao L. (2021). A global database of COVID-19 vaccinations. Nature Human Behaviour.

[bib25] McAteer J., Yildirim I., Chahroudi A. (2020). The VACCINES act: Deciphering vaccine hesitancy in the time of COVID-19. Clinical Infectious Diseases.

[bib26] Nivette A., Ribeaud D., Murray A., Steinhoff A., Bechtiger L., Hepp U., Shanahan L., Eisner M. (2021). Non-compliance with COVID-19-related public health measures among young adults in Switzerland: Insights from a longitudinal cohort study. Social Science & Medicine.

[bib27] Nomura S., Eguchi A., Yoneoka D., Kawashima T., Tanoue Y., Murakami M., Sakamoto H., Maruyama-Sakurai K., Gilmour S., Shi S., Kunishima H., Kaneko S., Adachi M., Shimada K., Yamamoto Y., Miyata H. (2021). Reasons for being unsure or unwilling regarding intention to take COVID-19 vaccine among Japanese people: A large cross-sectional national survey. The Lancet Regional Health – Western Pacific.

[bib28] Ogilive G.S., Gordon S., Smith L.W., Albert A., Racey C.S., Booth A., Gottschlich A., Goldfarb D., Murray M.C.M., Galea L.A.M., Kaida A., Brotto L.A., Sadarangani M. (2021). Intention to receive a COVID-19 vaccine: Results from a population-based survey in Canada. BMC Public Health.

[bib29] Rouw A., Wexler A., Kates J., Michuad J. (2021). https://www.kff.org/coronavirus-covid-19/issue-brief/tracking-global-covid-19-vaccine-equity/.

[bib30] Schwarz J.L., Caplan A.L. (2011). Vaccination refusal: Ethics, individual rights, and the common good. PrimaryCare.

[bib31] Soares P., Rocha J.V., Moniz M., Gama A., Laires P.A., Pedro A.R., Dias S., Leite A., Nunes C. (2021). Factors associated with COVID-19 vaccine hesitancy. Vaccines.

[bib32] The Government of the Hong Kong Special Administrative Region (2021). https://www.covidvaccine.gov.hk/en/dashboard.

[bib33] The Government of the Hong Kong Special Administrative Region (2022). https://www.chp.gov.hk/files/pdf/local_situation_covid19_en_20220209.pdf.

[bib34] The Organisation for Economic Co-operation and Development (2021). Enhancing public trust in COVID-19 vaccination: The role of governments. https://www.oecd.org/coronavirus/policy-responses/enhancing-public-trust-in-covid-19-vaccination-the-role-of-governments-eae0ec5a/.

[bib35] Tré-Hardy M., Cupaiolo R., Wilmet A., Antonine-Moussiaux T., Vecchia A.D., Horeanga A., Papleux E., Vekemans M., Beukinga I., Blairon L. (2021). Immunogenicity of mRNA-1273 COVID vaccine after 6 months surveillance in health care workers: A third dose is necessary. Journal of Infection.

[bib36] Viswanath K., Bejalu M., Dhawan D., Pinnamaneni R., Lang J., McLoud R. (2021). Individual and social determinants of COVID-19 vaccine uptake. BMC Public Health.

[bib37] Wang J., Jing R., Lai X., Zhang H., Lyu Y., Knoll M.D., Fang H. (2020). Acceptance of COVID-19 vaccination during the COVID-19 pandemic in China. Vaccines.

[bib38] Wang K., Wong E.L.Y., Ho K.F., Cheung A.W.L., Yau P.S.Y., Dong D., Wong S.Y.S., Yeoh E.K. (2021). Changes of willingness to accept COVID-19 vaccine and reasons of vaccine hesitancy of working people at different waves of local epidemic in Hong Kong, China: Repeated cross-sectional surveys. Vaccines.

[bib39] Ward P.R. (2017). Improving access to, use of, and outcomes from public health programmes: The importance of building and maintaining trust with patients/clients. Frontiers in Public Health.

[bib40] van der Weerd W., Timmermans D.R., Beaujean D.J., Oudhoff J., van Steenbergen J.E. (2011). Monitoring the level of government trust, risk perception and intention of the general public to adopt protective measures during the influenza A (H1N1) pandemic in The Netherlands. BMC Public Health.

[bib41] Weinstraub R.L., Subramanian L., Karlage A., Ahmad I., Rosenberg J. (2021). COVID-19 vaccine to vaccination: Why leaders must invest in delivery strategies now. Health Affairs.

[bib42] Wong M.C.S., Wong E.L.Y., Huang J., Cheung A.W.L., Law K., Chong M.K.C., Ng R.W.Y., Lai C.K.C., Boon S.S., Lau J.T.F., Chen Z., Chan P.K.S. (2021). Acceptance of the COVID-19 vaccine based on the health belief model: A population-based survey in Hong Kong. Vaccine.

[bib43] World Health Organization (2021). https://www.who.int/news/item/26-11-2021-classification-of-omicron-(b.1.1.529)-sars-cov-2-variant-of-concern.

[bib44] World Health Organization (2021). https://www.who.int/publications/m/item/draft-landscape-of-covid-19-candidate-vaccines.

[bib45] World Health Organization (2021). WHO Coronavirus (COVID-19) dashboard. https://covid19.who.int/.

[bib46] World Health Organization (2021). https://www.who.int/news-room/spotlight/ten-threats-to-global-health-in-2019.

[bib47] Wu Y.S., Wang M.P., Ho S.Y., Cheung Y.T., Kwong A., Lai V., Lam T.H. (2019). Positive perceptions of electronic cigarettes relative to combustible cigarettes are associated with weaker support for endgame policies on combustible cigarettes: A population-based cross-sectional study in Hong Kong. Tobacco Induced Diseases.

[bib48] Xu Z., Liu K., Gao G.F. (2022). Omicron variant of SARS-CoV-2 imposes a new challenge for the global public health. Biosafety and Health.

[bib49] Zhao S.Z., Wang M.P., Viswanath K., Lai A., Fong D.Y.T., Lin C.C., Chan S.S.C., Lam T.H. (2019). Short sleep duration and insomnia symptoms were associated with lower happiness levels in Chinese adults in Hong Kong. International Journal of Environmental Research and Public Health.

